# Datasets assessing lipid-content in optically cleared brains

**DOI:** 10.1016/j.dib.2023.109795

**Published:** 2023-11-30

**Authors:** Shimrit Oz, Galit Saar, Shunit Olszakier, Ronit Heinrich, Mykhail O. Kompanets, Shai Berlin

**Affiliations:** a Department of Neuroscience, Faculty of Medicine, Technion-Israel Institute of Technology, Haifa, Israel; b Biomedical Core Facility, Faculty of Medicine, Technion-Israel Institute of Technology, Haifa, Israel; c L.M. Litvinenko Institute of Physico-Organic Chemistry and Coal Chemistry, National Academy of Sciences of Ukraine, Kyiv, Ukraine

**Keywords:** Tissue-clearing, Lipids, MRI, Imaging, Histochemistry, Fluorescent probe

## Abstract

Multi-modal imaging, by light-microscopy (LM) and Magnetic Resonance Imaging (MRI), holds promise for examining the brain across various resolutions and scales. While MRI acquires images in three dimensions, acquisition of intact whole-brain by LM requires a process of tissue clearing that renders the brain transparent. Removal of lipids (delipidation) is a critical step in the tissue clearing process, and was previsouly suggested to be the cause for absence of MRI contrast in cleared brains. Yet, the association between MRI contrast, delipidation and the different clearing techniques is debatable. Here, we provide datasets concerning lipid-content in cleared brain tissues obtained by various approaches. Fixed mouse and rat brains were cleared by CLARITY, Sca*l*e, uDISCO and ECi clearing techniques. Lipid-content was assessed at various intermediate steps of the different clearing methods, as well as at the end of the processes. Methods employed included whole brain MRI acquisition, Oil Red O (ORO)- and carbocyanine DiI-staining of cryosections, and DiI-washout assay from brain slices. MRI contrast-to-noise ratio, staining intensities and integrity of tissue were systematically analyzed. We demonstrate that lipid electrophoresis, an essential step of the CLARITY approach, engenders progressive reduction in MRI contrast in non-cleared (PFA-fixed) control brains, as well as strongly reduces contrast from uDISCO and ECi-cleared brains. ORO minimally stained CLARITY-cleared brains, however efficiently labelled uDISCO and ECi-cleared brains. Conversely, and in contrast to ORO-staining, DiI equally stained control, CLARITY, ECi and uDISCO-cleared brains. Both ORO- and DiI-staining demonstrated impairment in brain tissue integrity following CLARITY, but less so in uDISCO and ECi brains. DiI-washout assay demonstrated that each of the solvents employed along the process of uDISCO and ECi are highly delipidating, as well as the SDS-electrophoresis employed during CLARITY clearing. However, Sca*l*e treatment preserved most of the DiI dye. These data emphasize the variability in lipid assessment of cleared tissues by common techniques, and may help to resolve the contribution of lipids in brain MRI contrast.

Specifications Table

**Table:** 

Subject	Molecular neuroscience: General
Specific subject area	Lipid assessment following tissue clearing
Data format	Raw Analysed
Type of data	Images, Graphs
Data collection	Whole brains were scanned with 9.4T MRI (Bruker Biospec, Ettlingen, Germany) interface with Avance III console, equipped with a cylindrical transmit volume coil (86 mm inner diameter) and a surface coil (20 mm diameter). Cryosections were imaged using automated slide scanner (Panoramic 250 Flash III, 3D Histech LTD). Snapshots of brains and brain slices (placed on a 0.5 cm grid paper) were taken by a regular hand-held digital camera.
Data source location	Institution: Technion - Israel Institute of Technology City/Town/Region: Haifa/Bat Galim/North Country: Israel
Data accessibility	Raw data and images can be found under repository name: 1- DiI staining of brains following clearing protocols; DOI:10.17632/6f3y72tdxt.1; Direct URL to data: https://data.mendeley.com/datasets/6f3y72tdxt/1 2- ORO staining of brains following clearing protocols; DOI:10.17632/gjwr2dtrrp.1; Direct URL to data: https://data.mendeley.com/datasets/gjwr2dtrrp/1
Related research article	no

## 1 Value of the Data

• These data-sets allow the reader to appreciate the unsuitability, variability and potential errors that may arise when trying to assess or quantify lipid-content by common methods in brain tissues that have been optically-cleared by various chemical methods.
• Our results demonstrate the technical hurdles when handling cryosections of cleared brains.
• Our data can be used for comparing histological data between tissues that have been cleared by various methods.

## 2 Data Description

**Fig. 1:**
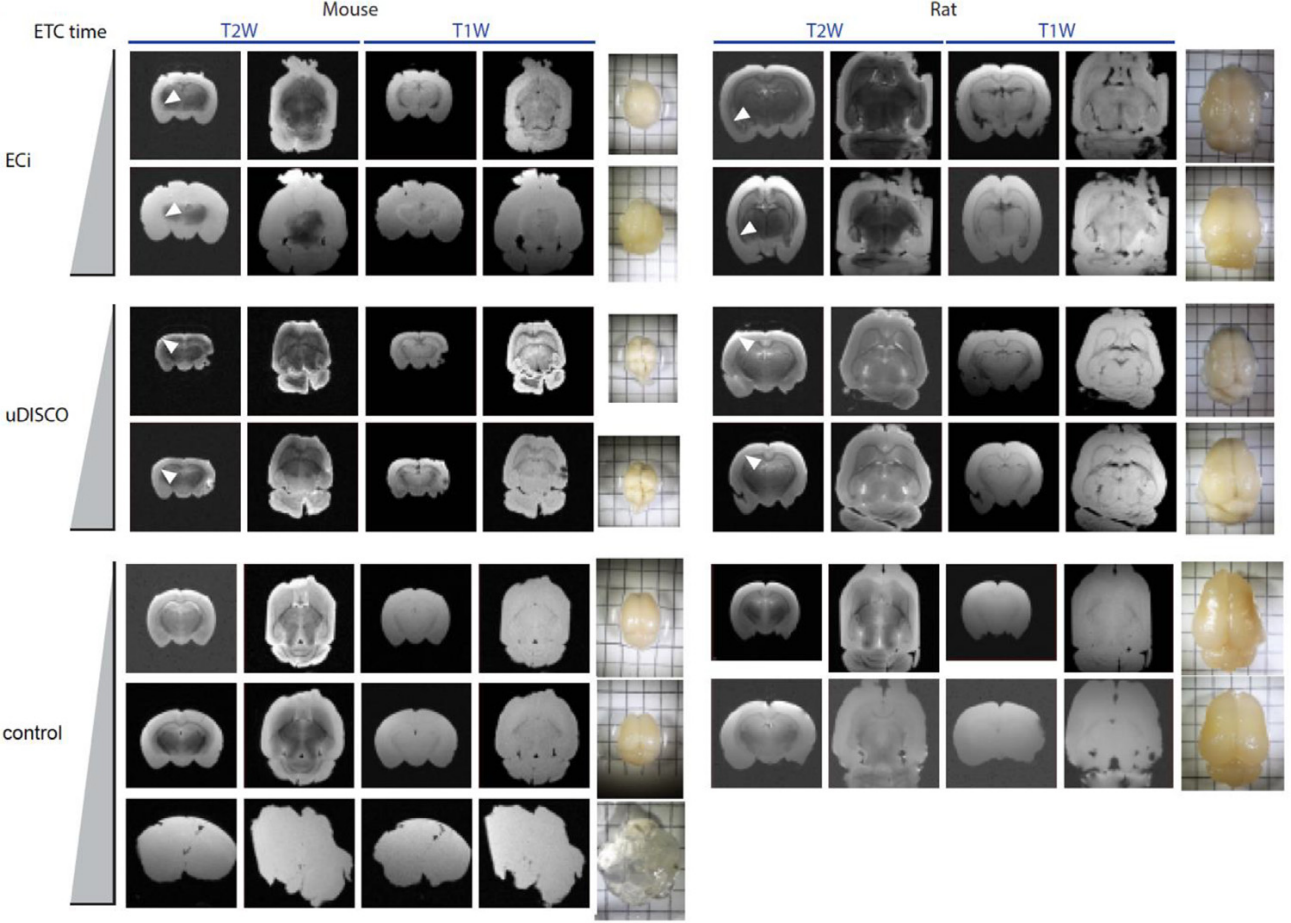
ETC gradually reduces MRI contrast. Rat and mouse brains treated for six and 24 h by ETC, after clearing by ECi and uDISCO protocols. Non-treated control brains were similarly treated by ETC for six and 24 h, in addition to 36 h for a mouse brain. Coronal and horizontal slices T1W and T2W MR-images, and snapshot of samples on a 0.5 cm grid paper, are shown. White arrows demonstrate progressive loss of MRI.

In a large set of experiments, we assessed lipid-content in rodent brains after these have been, supposedly, delipidated by multiple tissue-clearing techniques. The clearing techniques examined involve multiple delipidation steps; each of which was assessed for its ability to delipidate the tissue. Lipid-content was examined by MRI (to assess MRI-contrast), and by lipid-staining techniques. We employed solvent-based tissue-clearing techniques, explicitly ECi [1] and uDISCO [2], and hydrophilic techniques, such as CLARITY [3] and Sca*l*e [4]. We first examined whether we could observe contrast in MRI images. Notably, previous reports suggest that lipids (such as in myelin and membranes), are a dominant source for MRI-contrast, specifically in brain tissue [5]. Therefore, delipidating tissue-clearing methods are expected to eliminate contrast in cleared tissues. However, we found that the delipidating methods uDISCO and ECi yield cleared-whole brains that do show MRI-contrast in the tissue. These suggested incomplete removal of lipids from the cleared specimen by the latter approaches. We then aimed to further delipidate uDISCO- and ECi- brains by active electrophoretic tissue-clearing (ETC) system; a system that elutes lipids by an electric field in a SDS-containing solution, as is done during CLARITY-clearing. We noted that ETC-treatment prompted expansion of uDISCO and ECi-brains, combined with changes in color (shift towards yellowish-white), increased opaqueness, and changing tissue consistency towards a more gelatinous state (Fig. 1). Of note, changes in features of the tissue were mainly apparent in the outer layers of the brain, with noticeable “unperturbed” interiors. Correspondingly, we observed non-uniform reductions in MRI-contrast at these same outer layers— showing pronounced reductions in MRI-contrast the longer ETC was applied (seen as bright and swollen edges in the outer layers of the brain; Fig. 1, **arrowheads**). ECi-cleared brains were more readily affected by the ETC-treatment than uDISCO-cleared brains, and this was also reflected by a stronger reductions in MRI-contrast in ECI- than uDISCO-cleared brains. Control brains (i.e., PFA-fixed brains that were kept in PBS) showed the strongest reductions (i.e, complete loss) in MRI-contrast following prolonged ETC-treatment (tissues were also very fragile and deformed) (Fig. 1).

**Fig. 2:**
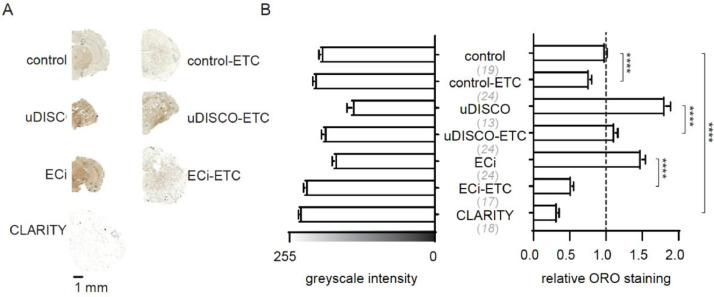
ORO-lipid staining of cleared mouse brain cryosections. A. Representative ORO-stained cryosections. B. (left) Summary of ORO-staining (greyscale intensities) of cryosections following the different clearing treatments, and (right) relative staining (the intensity of each cryosections was normalized to the average intensity of the control group in each experiment). Number of sections is noted in brackets. *n*= 4 independent experiments.

We then proceeded to compare lipid-content in cleared tissues using standard lipid-staining techniques. We stained cryosections of cleared-brains with Oil Red O (ORO). ORO is a fat-soluble diazol-dye commonly employed to detect neutral lipids, lipid droplets and cholesteryl esters, but not biological membranes [6,7]. We initially validated the method by comparing the staining of control (PFA-fixed) and CLARITY brains as positive and negative controls, respectively. CLARITY cryosections showed very weak ORO-staining as expected [8], whereas control brains showed positive staining (Fig. 2). Furthermore, ORO-staining could be reduced from control brains by prolonged ETC-treatment (Fig. 2, **control-ETC**). Conversely, cryosections from uDISCO- and ECi-brains showed extensive ORO-staining; higher than in the positive control, and this staining could be reduced by subsequent ETC-treatment (Fig. 2).

**Fig. 3:**
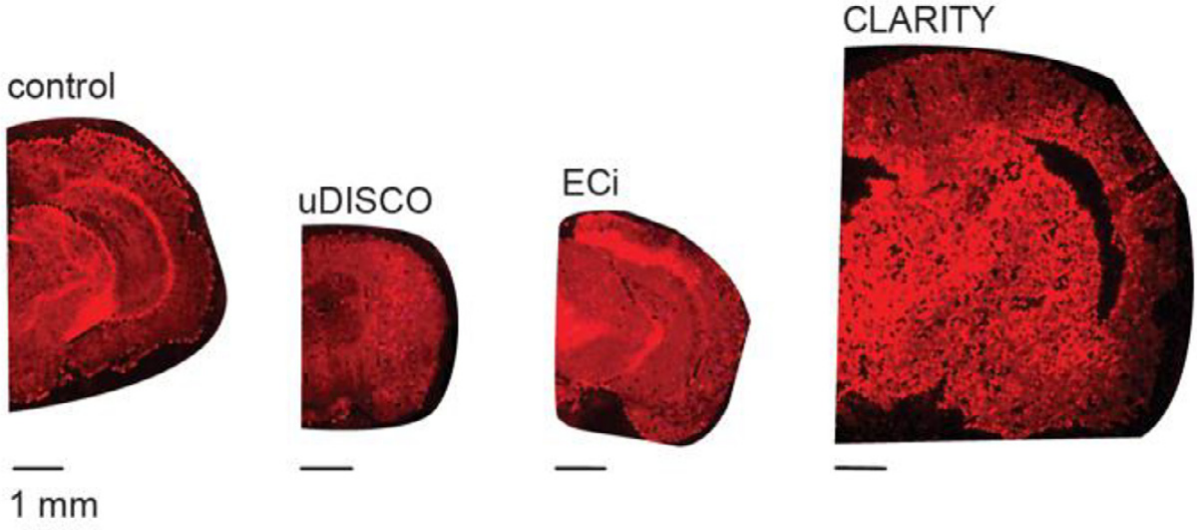
DiI-staining of cleared mouse brain cryosections. Representative images of stained cryosection showing the red fluorescence of the dye. Treatments are noted above sections. Bar- 1 mm. Note the change in size of the sections and the sponge like appearance of CLARITY-sections.

To test for phospholipid-content, we employed 1,1′-dioctadecyl-3,3,3′,3′-tetramethylindocarbocyanine perchlorate (DiI). DiI is a lipophilic dye that intercalates into phospholipids, therefore is used to label cellular membranes [9] and, importantly, has been previously employed to assess lipids in cleared samples [4,8,[10], [11], [12]]. Nevertheless, when applied onto CLARITY, uDISCO or ECi-cleared brains cryosections, DiI labeled all sections to a similar extent as controls (Fig. 3), negating the above mentioned results (see Fig. 2). When examining the integrity of cleared tissues, both DiI and ORO staining demonstrate the deleterious effect of the CLARITY procedure over tissues (rendering it highly porous), compared to control, ECi- and uDISCO-brains sections (Figs. 2 and 3).

**Fig. 4:**
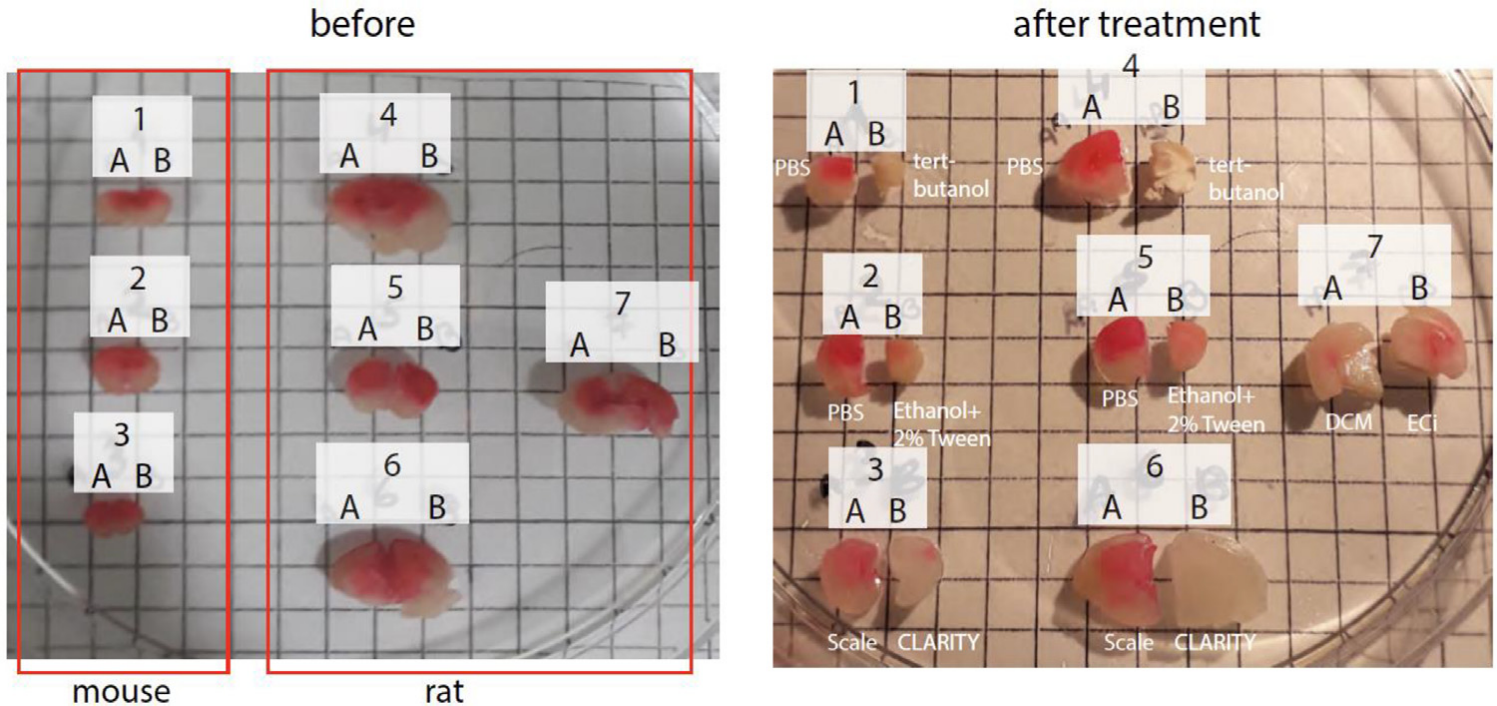
DiI-washout assay. DiI (for labeling of phospholipids) was applied onto PFA-fixed intact brains (onto each hemisphere) of mouse (Samples 1–3) and rat brains (4–7). Brains were then sectioned in the midline (yielding A and B sections) (left images, ‘before’) followed by treatment (right images, after treatment) by various reagents of the different clearing protocols: Samples 1A and 4A were immersed in PBS, whereas their counterparts (samples 1B and 4B) were immersed in tert-butanol for four days at 35 °C. Samples 2A and 5A were immersed in PBS, whereas 2B and 5B in ethanol + 2 % tween for four days at 4 °C. Samples 3A and 6A were immersed in the Sca*l*e solution for seven days at RT, and 3B and 6B treated by CLARITY (including three hours ETC). Samples 7A and 7B were immersed for one hour at RT in DCM and ECi solution, respectively. grid- 0.5 cm.

We further employed a ‘DiI washout assay’, wherein thick (2 and 3 mm) PFA-fixed coronal brain slices were pre-stained with a drop of DiI then treated by different clearing techniques to examine their de-staining capacities (Fig. 4). DiI was completely washed-out following the CLARITY protocol, suggesting that this procedure can remove lipids efficiently (Fig. 4, **slices 3B, 6B**). This is consistent with ORO-staining, but not with the DiI-staining of CLARITY specimen. We also examined the various clearing steps in uDISCO and ECi methods and observed that the dehydration steps (*tert*-butanol and ethanol, and 2 % Tween-20, respectively) are also strongly delipidating as in CLARITY, yielding significantly reduced DiI-staining with *tert*-butanol exhibiting more pronounced DiI-destaining capacity (Fig. 4**, slices 1, 2, 4, 5**). These are also in striking contrast to results obtained by the ORO- and DiI-staining of uDISCO and ECi-brains. The main delipidating reagents in uDISCO and ECi (i.e., Dichloromethane; DCM, and ECi, respectively) are also efficient destaining agents (Fig. 4**, slice 7**). As an additional control, we have also analyzed a lipid-retaining tissue-clearing process, denoted Sca*l*e [4]. Sca*l*e achieves optical transparency by hyperhydration of the tissue. Indeed, Sca*l*e engendered very gentle destaining of DiI from brains tissues (Fig. 4**, slices 3A, 6A**).

In summary, CLARITY shows minimal staining when assessed by ORO and DiI-washout assay, but not by DiI-staining (Figs 2–4). ETC is associated with reduction in MRI contrast (Fig. 1). These argue againstroles of lipids in MRO contrast [5]. However, ORO and DiI-staining of ECi and uDISCO-brains suggests retention of lipids, despite the notion that these should be delipidating approaches (Figs. 2 and 3). Conversely, the DiI-washout assay (i.e., assessment of DiI-destaining by the clearing procedure) demonstrates that both ECi and uDISCO readily remove phospholipids, while Sca*l*e technique preserves lipids (Fig. 4).

## 3 Experimental Design, Materials and Methods

**Animals:** We used three-weeks to 6 months old, male and female, C57BL/6 mice and Sprague Dawley rats (Envigo, Israel). Animals were transcardially perfused with PBS (02-023-5A, Biological Industries) and neutral buffered 10 % formalin (HT5011, Sigma-Aldrich). Then, fixed-brains were removed and placed in formalin at 4 °C for 24 h, washed and incubated in PBS at 4 °C until clearing, cryosectioning or imaging.

**CLARITY**[3]**:** The procedure was performed using a commercial CLARITY-clearing system and reagents (X-CLARITY; Logos Biosystems). Whole brains (Figs 2 and 3) and slices (Fig. 4) were infused with hydrogel monomers at 4 °C overnight (cat #: C1310X, Logos Biosystems) and a subsequent hydrogel polymerization step was performed at 37 °C for three hours at -90 kPa (cat #: C20001, Logos Biosystems). Polymerized brains were then actively cleared in electrophoretic tissue clearing (ETC) solution containing 200 mM boric acid and 4 % (wt/vol) SDS (pH 8.5) (C13001, Logos Biosystems) by a Tissue-Clearing System (cat #: C10001, Logos Biosystems). Whole mouse brains were placed in the electrophoretic chamber under constant current of 1.2 A for 6 h, whereas whole rat brains at 1.2 A for 10 h. ETC of CLARITY slices (Fig. 4) was only for 3 h.

**ETC:** ECi and uDISCO cleared brains were hydrated in PBS (overnight, shaking at RT) prior to ETC. Brain samples were transferred into the ETC chamber (in ETC solution) at 1.2 A, 37 °C for 12 h (Fig. 2) or as indicated (6, 24 and 36 h as indicated, Fig. 1).

**uDISCO**[2]: Whole brains (Figs 1-3) were dehydrated by sequential immersion in incrementing concentrations of *tert*-butanol (30, 50, 70, 80, 90, 96, and 100 % vol in DDW; cat #: 360538, Sigma-Aldrich) for 4–12 h in each solution along gentle shaking at 34 °C. Then, samples were delipidated by incubation in Dichloromethane (DCM; cat #: 270997, Sigma-Aldrich) for 1 and 2 h at RT.

**ECi**[1]: Whole brains (Figs 1–3) were dehydrated by sequential immersion in incrementing concentrations of ethanol (30, 50, 70, 100 % vol in DDW; 30–70 % solutions were adjusted to pH9) supplemented with 2 % Tween-20 (cat #: P1379, Sigma-Aldrich) for 12–24 h in each solution with gentle shaking at 4 °C. Then, samples were delipidated in 100 % ethyl-cinnamate (ECi, cat #: 112372, Sigma-Aldrich) for at least 24 h, until desired transparency is obtained.

**Scale**[4]: Brain slice was immersed in Sca*l*e-A2 solution containing: 4 M urea (cat #: U5128, Sigma-Aldrich), 10 % (v/v) glycerol (cat #: 191612, Sigma-Aldrich) and 0.1 % (v/v) Triton X-100 (cat #: X100, Sigma-Aldrich) adjusted to pH 7.6, with gentle shaking at RT.

**Magnetic resonance imaging (MRI) acquisition:** Images were acquired using a 9.4T MRI system horizontal bore system (Bruker Biospec, Ettlingen, Germany) interface with Avance III console; equipped with a cylindrical transmit volume coil (86 mm inner diameter) and a surface coil (20 mm diameter) for detection. Brains were placed in cylindrical tubes and immersed in fomblin (fomblin-Y LVAC 06/6, cat #: 317926, Sigma-Aldrich) to minimize susceptibility artifacts. MRI protocols included coronal and horizontal anatomical T2W and T1W scans. T2W images were acquired using a Rapid Acquisition with Relaxation Enhancement sequence (RARE), at 0.4 (mouse) and 0.6 (rat) mm slice thickness of 32 Slices, 100 µm in plane resolution with the following features: TR = 3500–4110 ms, TE = 36 ms, RARE factor = 12, FOV = 1.6 × 1.6 cm^2^ (mouse) or 1.92 × 1.92/2.2 cm^2^(rat), matrix size = 160×160 (mouse) or 192×192/220 (rat), number of averages = 6. T1W images were acquired with a fast low angle shot (FLASH) sequence with the same geometry and resolution as T2W sequences, and with TR/TE = 320/4 ms, 30^o^ pulse, number of averages = 6. Data processing was performed using Medical Image Processing Analysis, and Visualization (MIPAV) software (NIH).

**Cryosections of fixed and cleared samples:** Control and cleared brains were sequentially immersed in 15, 20 and 30 % sucrose in PBS, for 12 h in each solution at 4°C. Brains were then embedded in optimal cutting temperature (OCT) media and frozen at -80°C. Coronal sections (30 µm thick) were sectioned by a cryostat, and mounted on microscope slides (SuperFrost Plus, Thermo Scientific).

**Lipid staining:** For ORO staining, cryosections (mounted on microscope slides) were immersed in 0.3 % ORO in 60 % isopropanol (cat #: O0625, Sigma-Aldrich) and incubated for 12 h at RT, then washed three times in PBS. Sample size (number of cryosections) for ORO treatment: control *n*=19 (from 6 brains), control ETC *n*=24 (from 8 brains), ECi *n*=24 (from 8 brains), ECi-ETC *n*=17 (from 6 brains) uDISCO *n*=13 (from 4 brains), uDISCO-ETC *n*=24 (from 8 brains) CLARITY *n*=18 (from 5 brain). *n*=4 staining experiments, a detailed summary is in the .xlsx file. For DiI staining, cryosections were immersed in 5 µg/ml DiI (cat #: 42364, Sigma-Aldrich), for two hours followed by washing with PBS. Slides were sealed with mounting glue (ImmuMount, Thermo scientific) on coverslips, and scanned by an automated slide scanner (Pannoramic 250 Flash III, 3D Histech LTD). Sample size (cryosections) for DiI treatment (*n*=3 staining experiments): control *n*=9 (from 3 brains), control-ETC *n*=6 (from 2 brains), ECi *n*=14 (from 4 brains), uDISCO *n*=3 (from 1 brain), uDISCO-ETC *n*=3 (from 1 brain), CLARITY *n*=11 (from 4 brains). Low number of uDISCO brain samples was due to the difficulty to section and adhere to cover-glass (see section “Limitations”). Histological (Fig. 2) and fluorescent (Fig. 3) images were converted to 32-bit greyscale, artifacts and background were excluded, and intensity was calculated for each image (ImageJ, NIH). For relative ORO staining, the grey intensity from each slice was normalized to the average grey intensity in images from PBS control group, in each independent experiment.

**DiI-washout assay**: 1 µl of DiI-solution (1 mg/ml in PBS) was pipetted onto 3 mm thick hemispheres from coronal brain slices and was absorbed in the tissue for 1 h. The “stained” hemispheres were incubated in different tissue-clearing solutions, as described for each clearing procedure, e.g. treatment with tert-butanol and Ethanol+Tween included a sequential immersion in incrementing concentrations. The slice treated with Sca*l*e, was immersed for 7 days in Sca*l*e solution. Controls (PFA-fixed, samples 1A, 2A, 4A, 5A) were maintained in PBS under identical conditions (temperature, shaking rate and incubation times) as their treated counterpart hemispheres.

## Limitations

Unwashed ECi and uDISCO-cleared brains disintegrated during cryosectionning, whereas extensive washing of the brains with sucrose in PBS facilitated sectioning and preserved tissue integrity. In addition, CLARITY-cleared brains were challenging to section at <30 µm, and sections made from uDISCO-cleared brains that underwent extensive washing by PBS proved difficult to adhere to the microscope slides.

## Funding

Support was provided by the 10.13039/501100000781European Research Council starting-grant (SB, ERC-stg-851919).

## Ethics Statement

All animal procedures were in accordance with the guidelines and regulations of the Technion and were approved by the Animal Care and Use Committee of the Technion - Israel Institute of Technology (Haifa, Israel, Ethic number IL-121-08-19).

## CRediT authorship contribution statement

**Shimrit Oz:** Conceptualization, Methodology, Writing – original draft. **Galit Saar:** Data curation. **Shunit Olszakier:** Investigation. **Ronit Heinrich:** Investigation. **Mykhail O. Kompanets:** Investigation. **Shai Berlin:** Writing – original draft, Conceptualization, Supervision.

## Declaration of Competing Interest

The authors declare that they have no known competing financial interests or personal relationships which have or could be perceived to have influenced the work reported in this article.

## Data Availability

2-ORO staining of brains following clearing protocols (Original data) (Mendeley Data). 1-DiI staining of brains following clearing protocols (Original data) (Mendeley Data).
